# Professional Experiences and Career Trajectories of Mid- to Senior-Career Women Clinician-Scientists: A Qualitative Study

**DOI:** 10.1001/jamanetworkopen.2024.6040

**Published:** 2024-04-11

**Authors:** Lauren A. Szczygiel, Amanda K. Greene, Christina M. Cutter, Rochelle D. Jones, Eva L. Feldman, Kelly C. Paradis, Isis H. Settles, Kanakadurga Singer, Nancy D. Spector, Abigail J. Stewart, Peter A. Ubel, Reshma Jagsi

**Affiliations:** 1 Department of Surgery, University of Michigan Medical School, Ann Arbor; 2 Center for Bioethics and Social Sciences in Medicine, University of Michigan Medical School, Ann Arbor; 3 Department of Emergency Medicine, University of Michigan Medical School, Ann Arbor, Michigan; 4 Department of Radiation Oncology, Emory University, Atlanta, Georgia; 5 Department of Neurology, University of Michigan Medical School, Ann Arbor; 6 Department of Radiation Oncology, University of Michigan Medical School, Ann Arbor; 7 Department of Psychology, University of Michigan, Ann Arbor; 8 Department of Afroamerican and African Studies, University of Michigan, Ann Arbor; 9 Department of Women’s and Gender Studies, University of Michigan, Ann Arbor; 10 Department of Pediatrics, University of Michigan Medical School, Ann Arbor; 11 Department of Pediatrics, Drexel University College of Medicine, Philadelphia, Pennsylvania; 12 Duke University School of Medicine, Durham, North Carolina

## Abstract

**Importance:**

Despite increasing evidence and recognition of persistent gender disparities in academic medicine, qualitative data detailing the association of gender-based experiences with career progression remain sparse, particularly at the mid- to senior-career stage.

**Objective:**

To investigate the role gender has played in everyday professional experiences of mid- to senior-career women clinician-scientists and their perceptions of gender-related barriers experienced across their careers.

**Design, Setting, and Participants:**

In this qualitative study, a total of 60 of 159 invited clinician-scientists who received National Institutes of Health K08 or K23 awards between 2006 and 2009 and responded to a survey in 2021 agreed to participate. Invitees were selected using random, purposive sampling to support sample heterogeneity. Semistructured in-depth interviews were conducted January to May 2022. For this study, interviews from 31 women were analyzed using the framework approach to thematic analysis. Data analyses were performed between August and October 2023.

**Main Outcomes and Measures:**

Descriptive themes of participant experiences of gender and gender-based barriers in academic medicine.

**Results:**

A total of 31 women clinician-scientists (8 identifying as Asian [25.8%], 14 identifying as White [45.2%], and 9 identifying as members of a minority group underrepresented in medicine [29.0%]; 14 aged 40-49 years [45.2%] and 14 aged 50-59 years [45.2%]) were included. Among them, 17 participants (54.8%) had children who required adult supervision or care, 7 participants (22.6%) had children who did not require supervision or care, and 6 participants (19.4%) did not have children. There were 4 dominant themes identified within participant experiences in academic medicine: the mental burden of gendered expectations at work and home, inequitable treatment of women in bureaucratic processes, subtle and less subtle professional exclusion of women, and value of communities built on shared identities, experiences, and solidarity.

**Conclusions and Relevance:**

This study found that women perceived the institution of academic medicine as a male-centric system misaligned with the needs of women, with associated feelings of exclusion, disillusionment, and loss of trust in their institutions. Findings suggest that the confluence of domestic obligations and unaccommodating institutional environments may make it difficult for women clinician-scientists to achieve established timelines of career progression and productivity; these findings may have long-term implications for the well-being and retention of women in academic medicine.

## Key Points

**Question:**

How have women mid- to senior-career clinician-scientists experienced gender throughout their careers?

**Findings:**

In this qualitative interview study among 31 women clinician-scientists, participants experienced the institution of academic medicine as a male-centric system misaligned with the needs of women and people with family caregiving responsibilities. They felt that women’s needs were underrecognized and unaccommodated even in the mid- to senior-career stage.

**Meaning:**

These findings suggest that structural inequities embedded in policies, processes, culture, and norms of academic medicine may contribute to well-documented patterns of gender disparities in career advancement that persist across women’s careers.

## Teaser

This qualitative study investigates mid- to senior-career women clinician-scientists’ perceptions of gender-related barriers and the role of gender in their professional experiences.

## Introduction

Despite long-standing awareness of gender inequities in academic medicine and implementation of targeted interventions, men remain overrepresented across multiple traditional markers of career success.^1^ These include but are not limited to publication productivity,^2^ acquisition of federal grant funding,^3^ and attainment of senior ranks^4,5^ and leadership positions.^6^ Research suggests that this observed inequity may be in part attributed to gender-based discrimination, bias, and harassment.^7,8^ While overt harassment remains concerningly prevalent, more subtle forms of gender-based discrimination in the form of implicit bias or exclusion are even more pervasive. For example, a 2021 study^9^ points to a lack of transparency around tenure and promotion that can implicitly benefit men. Furthermore, women clinician-scientists often have substantially more domestic and parental responsibilities than men.^10,11^ Consequently, women are more likely than men to leave academic medicine or reduce their work hours after having children. Those who continue in academic medicine may also experience additional maternal discrimination.^12^

There are limited qualitative data on how women clinician-scientists experience the role of gender, particularly among women who have been in academic careers long enough to have reached the associate to full professor career stage.^6,13^ These mid- to senior-career women are astute observers of how gender operates within academic medicine, helping provide a nuanced view of the many interlocking factors that may contribute to continuing inequity in the field and their cumulative weight over time. Accordingly, this study was designed to address the following research questions: How do mid- to senior-career women in academic medicine perceive their gender influencing their everyday professional experiences and broader career trajectory? What gender-related barriers do these women perceive at this stage of their careers, and how do they mitigate the impact?

## Methods

### Study Design and Participants

This qualitative study received approval and exemption from ongoing review by the University of Michigan Institutional Review Board. Participants gave verbal informed consent. The study follows the Standards for Reporting Qualitative Research (SRQR) reporting guideline.

The research team was composed of a multidisciplinary team of researchers, including clinician-scientists with extensive lived experience in academic medicine, social scientists with qualitative methodological expertise, and others with complementary perspectives from varied clinical specialties and academic institutions. Our examination of gender-specific career experiences of women in academic medicine was guided by a social constructivist perspective, attributing differences between men and women to societal structures and interactions rather than intrinsic biological differences.^14^ This epistemological orientation centers individuals’ personal interpretation of their experiences when seeking to understand social phenomena.^15,16,17^

We recruited mid- to senior-career clinician-scientists for a qualitative study between January and May 2022. The data reported here are part of an ongoing longitudinal study of clinician-scientists who received National Institutes of Health (NIH) K08 or K23 career development awards between 2006 and 2009.^18^ We surveyed individuals 3 times, most recently in 2021.^19^

We invited participants from the survey who provided their race and ethnicity, gender, sexual orientation, and academic position. We stratified individuals by race and ethnicity (Asian, White, or minority group underrepresented in medicine), gender identity and sexual orientation (cisgender and heterosexual or lesbian, gay, bisexual, transgender, queer, intersex, and asexual [LGBTQIA]+), and status in academic medicine (still in academic medicine or left academic medicine). When surveyed, participants self-identified race and ethnicity using a list of categories from which they chose all that applied (American Indian or Alaska Native, Asian, Black or African American, Hispanic or Latino, Native Hawaiian or Other Pacific Islander, and White), none of these describes me, or I identify as “write-in.” The underrepresented race or ethnicity in medicine category was defined as self-reporting a race or ethnicity other than Asian or White (majority categories), including American Indian or Alaska Native, Black or African American, Hispanic or Latino, Middle Eastern or North African, Native Hawaiian or Other Pacific Islander, or mixed race or ethnicity. Race and ethnicity were assessed to permit inclusion of women expected to encounter the phenomenon of intersectionality.^20^ We randomly selected interview participants to seek diversity in responses and participant heterogeneity.

We purposely oversampled minority gender, race and ethnicity, and sexual orientation groups by aiming to recruit them at equal numbers as majority groups. We invited 159 survey respondents to participate in the qualitative study, and 60 individuals agreed to be interviewed (37.7% acceptance rate). Of the total interview participants, there were 28 men, 31 women, and 1 nonbinary individual. This study focuses on women’s narratives of their career experiences.

### Data Collection

We conducted interviews by phone or teleconferencing software. The semistructured interview guide covered a broad range of career experiences (Table 1). Additionally, at the beginning of the interview, we cued participants to share whether they thought any experiences they discussed were influenced by their personal identity characteristics. All interviews were audio recorded, transcribed verbatim, and deidentified.

**Table 1.** Interview Guide Domains and Example Questions

Topic domain	Example question
Introduction to interview topics	We’re going to spend this interview talking about your career and life experiences. Throughout the conversation, we’d like to be mindful of how your experiences reflect the unique intersection of your personal characteristics, including your gender, race and ethnicity, sexual orientation, religion, cultural background, and/or socioeconomic status….As we’re talking, please share with me if you think a certain perspective or experience was particularly influenced by any of your personal characteristics.
Current status	Tell me about some of the high points/low points of your career.
Giving and receiving mentorship	Thinking of all your mentors, colleagues, and trainees, have certain individuals played a memorable role in your career trajectory? Please talk about how certain individuals may have supported or impeded your career.
Experiences of civility and incivility	How much do you feel respect and that you fit in well at your institution/workplace? Do you feel like you’ve been treated differently in your career due to any of your personal characteristics or identities?
Well-being and work-life integration before and after COVID-19 pandemic	Prior to COVID, how well do you feel you were managing the responsibilities in your career and personal life?

### Data Analysis

We uploaded interview transcripts into MAXQDA 2022 software (VERBI Software) for storage and analysis. Our iterative, inductive analytic process was informed by the framework approach to thematic analysis (Figure).^21,22^ The coding framework was developed by L.A.S. and A.K.G. We reviewed transcripts from all women in the study and generated and refined codes until we reached coding saturation, the point at which no new codes were generated.^23^ The senior author (R.J.) and all other members of the study team provided feedback on the final coding scheme. After coding all transcripts, we reviewed the coded content, identified themes, and synthesized data across themes. Data analyses were performed between August and -October 2023.

**Figure.**
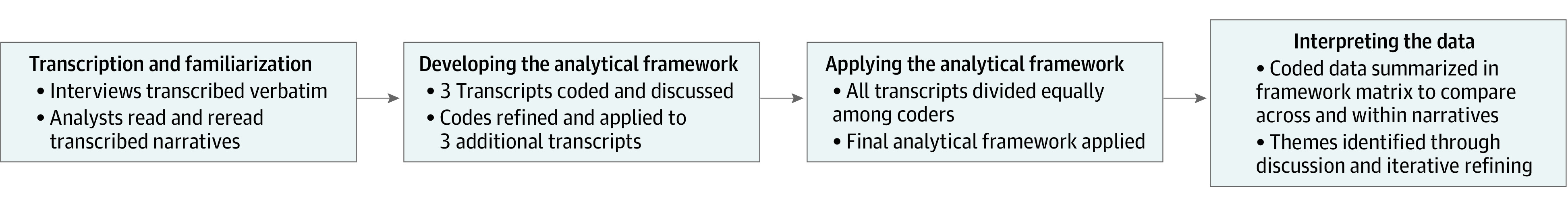
Data Analysis Informed by Framework Approach to Thematic Analysis

## Results

We interviewed 31 women clinician-scientists (8 identifying as Asian [25.8%], 14 identifying as White [45.2%], and 9 identifying as members of a minority group underrepresented in medicine [29.0%]; 14 aged 40-49 years [45.2%] and 14 aged 50-59 years [45.2%]) who received NIH K08 or K23 career development awards between 2006 and 2009 (Table 2). Among them, there was 1 assistant professor (3.2%), 11 associate professors (35.5%), 14 professors (45.2%), 1 professor emerita (3.2%), and 4 individuals who had left academic medicine (12.9%). There were 17 participants (54.8%) who had children who required adult supervision or care, 7 participants (22.6%) who had children who did not require supervision or care, and 6 participants (19.4%) did not have children. We identified 4 dominant themes, which are reported with exemplary quotations (Table 3), suggesting that the women in our sample experienced the institution of academic medicine as neither built nor evolved for the needs of women. Most women described subtle and at times less subtle ways in which they experienced academic medicine as “centered around male[s]” (participant 14).

**Table 2.** Demographics

Characteristic	Participants, No. (%) (N = 31)
Race and ethnicity^a^	
Asian	8 (25.81)
White	14 (45.16)
Minority group underrepresented in medicine	9 (29.03)
Women	31 (100)
Age in decades	
40s	14 (45.16)
50s	14 (45.16)
60s	3 (9.68)
Children status	
Has children who require adult supervision or care	17 (54.84)
Has children who do not require adult supervision or care	7 (22.58)
Does not have children	6 (19.35)
Missing	1 (3.23)
Academic rank	
Assistant professor	1 (3.23)
Associate professor	11 (35.48)
Professor	14 (45.16)
Professor emerita	1 (3.23)
Left academic medicine	4 (12.90)

^a^ Race and ethnicity has been categorized as Asian, White, and minority group underrepresented in medicine, which included everyone else (this category was defined as self-reporting a race or ethnicity other than Asian or White [majority categories], including American Indian or Alaska Native, Black or African American, Hispanic or Latino, Middle Eastern or North African, Native Hawaiian or Other Pacific Islander, or mixed race or ethnicity). Because multiple race and ethnicity answers could be selected, cases where this occurred were interrogated and preference given to the more minoritized group.

**Table 3.** Themes, Subthemes, and Representative Quotations of Women’s Experiences

Theme	Representative quotations
Theme 1: mental burden of gendered expectations at work and home	“…I think just the realities of the mental load and the mental burden that females carry with them. And that usually happens around midcareer, like late, early career…I just think can serve as a real challenge to women who are trying to navigate that successfully and kind of figure out how to do that.” Participant 4 (White woman) “[W]hen you think about balancing things, it means, oh, I can take something off of this side in order to give myself something else on the other side. There’s nothing you can take off. I can’t say…to my daughter…, ‘Well, I’m going to have to wait and do your hair on Thursday because that’s when it fits into my calendar.’ No, you just feel like sometimes you just are spread so thin….” Participant 30 (woman in minority group underrepresented in medicine) “Work-life balance is very hard. If you’re a female with kids, very, very hard. I see my other faculty with kids, it’s super hard. I see trainees with kids, it’s super hard. There’s no room in medicine if you have kids and you are female. You’re just stretched too thin.…And you have to really justify [needing to take time off]…because everybody has such a strong work ethic….That’s why the way I worked around it is I said, ‘I’m going part time.’…But again, that affects your career trajectory…” Participant 27 (Asian woman) “I think with COVID, one of the things that happened is when you’re at home all the time, it’s like I ended up working more, as many people did. I would get up early, and I would work before I got my son up.…After dinner I would work some more, and I worked way too much and I fully burned myself out. And then when you’re being told, again, you’re not working enough, that really just, it just stung, that ability to balance that was pretty hard when you felt like I should be working more.” Participant 55 (White woman) “[A] big decision for me to retire was to be able to spend more time with my family.…I didn’t miss much with my family [during my career], but I was always stressed.…Then, when my partner got into academia and the stresses were there for her as well, I don’t think we neglected our daughter, but I just didn’t feel like I was unstressed, ever, for 1 minute….**”** Participant 14 (White woman) “[T]here’s a big hit to productivity.…There’s a real, like, shift in priorities and values and time. And I mean, there’s just a lot that happens, I think, with having kids.” Participant 4 (White woman) “I just got emailed about [a grant opportunity].…I was like, ‘I just cannot even think about adding one more thing on my plate at this moment.’…Between having this big conference coming up and study section and grant writing and having a kid who’s going to college and working on editing her college essays when she asks me to, or all the things that go into that. I just could not envision adding even a single drop more on my plate.” Participant 11 (Asian woman) “I have triplets that are [young adults] and seniors in college….And if I could get all 3 of my kids into jobs in the next 6 months, that’s my real big issue. Or next stages in life.” Participant 2 (woman in minority group underrepresented in medicine) “[W]e’re in this funny generation where, again, our women mentors worked so hard to be successful and to break the glass ceiling. And then we showed up, and we’re supposed to continue all of that, but it’s like we’re being restricted. And all of the institutional barriers are still there. And again, you only have so much energy to fight against that when you’re never successful, and to see that these issues have been around and that they’re not going away and they’re not getting better, and that we’re grossly underpaid in our lifelong earnings. And not only that, but just the respect and how often our papers get published. And I want to be an agent of change, but boy, systemic issues.” Participant 28 (White woman)
Theme 2: inequitable treatment of women in bureaucratic processes	“The whole tenure process, everything about advancement at the university is centered around male[s].” Participant 14 (White woman) “[My] female colleagues had…gotten 2 [independent NIH awards]. My 1 male colleague had gotten [a similar NIH award]. And he had this suggestion that he should get standard milestone funding, but neither of my other [women] colleagues did....I went to the CEO and sort of said, ‘I would like standard milestone funding.…I don’t understand what I need to do to get standard milestone funding, but I’ve gotten [multiple independent NIH awards]’.…[T]hat left a really, really bad taste in my mouth…having a sense that there was inequity, but sort of uncovering it and having it be so blatant was really…challenging….[There’s] some myth around meritocracy that allows for a lot of inequities to persist around resource allocation.” Participant 8 (White woman) “…I got the K award funded, but I was taking...about a year and 3 months off of work. And I contacted my program officer at the NIH.…I basically said, ‘Can I defer getting the K award for a year because I’m not to be working on it?’ And they were basically like, ‘There’s no way to do that, so it’s just going to start....I understand you’re not going to be working this year…you’ll just apply for a no-cost extension when the grant is over. And so it’ll kind of instead of being a 5-year award because become a 6-year award.’…[I]n practical terms, [it] worked out fine, but it was also sort of annoying that the NIH didn’t have a mechanism to pause the grant or delay the grant when it was necessary to do so. That was absolutely the biggest obstacle in my career in terms of that.” Participant 17 (Asian woman) “That was another low point. I had a [mentored NIH award]. I had an [independent NIH award]. There’s a bunch of stuff that happened in that time where I didn’t immediately go up for promotion. I had [medical fertility issue]. I had young kids. I was trying to keep everything together. I didn’t get around to applying for promotion till very late.…And they didn’t forward my promotion application out of the department. They didn’t tell me for a year and then couldn’t tell me why. So I didn’t get promoted from assistant to associate for [more than 10] years.” Participant 16 (Asian woman) “[I] think men and women write grants very differently. And I suspect underrepresented minorities write grants more like women. Where we’re less confident to, say, to put out a statement that says, ‘This is how this will go,’ as opposed to, ‘This is maybe how it goes.’ And because of that, I think we struggle more in grant and study sections. Yeah, so it’s this idea of, well, study sections and science has been predominantly driven by White males who have a certain way of writing and doing things. And so that’s the footprint of what successful grants are.” Participant 11 (Asian woman) “[I]t’s quite frustrating to feel like you’ve done the things that you have needed to do but have not had the success of peers who haven’t done what we are supposed to do. You’re told, ‘Oh there’s a template. There are no surprises. You know what you need to do.’ Well, that is not true for certain people. It’s just not.” Participant 30 (woman in minority group underrepresented in medicine)
Theme 3: subtle and less subtle professional exclusion of women	“[M]y mentor…was very paternalistic. He would never let me meet with leadership on my own. He’s like, ‘Why don’t we meet together?’ Or, ‘I’ll take that to the department chair.’ And I’m like, ‘Why don’t I just go talk to him?’ And he’s like, ‘That’s not the way we do it here.’ And, so I was very much taught to sit down and shut up….” Participant 28 (White woman) “…I was like, ‘What do you mean he can’t meet with women on his own?’ And [my male senior colleague] said, ‘You cannot be in a room alone with him.’ And I was like, ‘You have got to be kidding.’ So this guy sexually harassed female colleagues, and they did nothing about it other than advise you to have a man in the room when you went.…They would tolerate what I thought was a horrid behavior just to keep people in and get the funding coming in.” Participant 29 (White woman) “And it was such a toxic environment. And on 1 hand, she was saying she was all about women and promoting women, and then the reality of it was she was screwing you over without even a second thought.” Participant 57 (White woman) “I think [gendered issues] are subtle, but in a way subtle ones are the most challenging....It can be even more challenging, because they set some sort of ceiling above your head that you may not even realize is there, right?…But is really shaping what you’re going to do with your career….[O]verall, it’s been more just the sense of being treated as a junior faculty member, not being invited to participate in senior leadership meetings, and things like that.” Participant 1 (White woman) “I feel like [my relationship with my mentor] somehow morphed into, like, this father-daughter thing rather than a scientific mentor–mentee situation, and I don’t feel like I was given guidance.…There just wasn’t scientific training. It was more like, ‘How are you going to feel fulfilled in life?’...In retrospect, this is not a very caring person who cares about how I progress in my career….This is some old dude worrying that some young woman is happy in life….You hear about [conversations with mentors] from men where they’re pissed off because, ‘The dude’s not going to let me graduate, and I got to redo this experiment or do more experiments,’…But the things you hear about that they have had conversations about have to do with the science, right? And when you talk to women, it’s like, ‘Oh, well, how are you going to set up time? When are you going to come and go and take care of your kid?’….I think the message…is that that should be a more important thing for you. You’re a woman.” Participant 16 (Asian woman) “So, there are committees and, especially as a woman and as a Latina, that you get asked to be on…if I just, like, had a disability, I would fill in 3 boxes for people because it gets to the point where you get asked to do things simply because of who you are…not because of what you bring to the table. And I mean, I’ve literally been told, ‘So, well, you’re young, you know, and you’re female, so we need you on the committee.’...[Y]ou never say that to a guy, like, you’re White and old, so we’ll put you on the committee.” Participant 5 (woman in minority group underrepresented in medicine)
Theme 4: value of communities built on shared identities, experiences, and solidarity	“I think the handful of events that [my institution] organized…around women in academic medicine, unfortunately the effect that they had was to convince me I needed to leave. But they were influential in supporting that I wasn’t the only one feeling this way and that I wasn’t isolated, that the shared experience of being a woman and was helpful because sometimes you feel crazy….” Participant 28 (White woman) “[W]e’ve been sort of taking some time with this committee to sort of create a call to action around equity and diversity issues in the [specialty] scientific workforce….The way that it resonates for me is maybe trying to take advantage of this opportunity that I’ve had, or this thing that I’ve experienced that I feel like other women in my career stage have also experienced and just tried to make sure it doesn’t happen for the women coming up behind us.” Participant 8 (White woman) “Some of our kids are a little older, and some are a little younger, but we all kind of shared parenting struggles with each other, work struggles with each other. We read books and we talked about books and drank wine and all that kind of stuff, but we really spent the majority of time just being together and listening to each other.…I think we gave ourselves confidence that we could do things.…We learned tricks and tips from each other, right? We learned, like, a couple of us shared babysitters, and we shared people who could help do your grocery shopping for you. We shared resources and ideas.” Participant 17 (Asian woman) “[The women] are just people who I really admire and respect and adore.…I could go to this group for all kinds of things, from just like a really bad day or getting something rejected that I really care about, to advice about how to strategize.…A big thing for me in my work-work balance is how much clinical time I’m doing versus how much time I have for a research. There definitely have been many negotiations with my boss over how much clinical time I should be doing.…We talked about all kinds of things, personal and professional.” Participant 13 (White woman)

Abbreviations: CEO, chief executive officer; NIH, National Institutes of Health.

### Theme 1: Mental Burden of Gendered Expectations at Work and Home

One of the most consistent and frequently mentioned themes in the interviews was the idea of trade-offs between career and family life due to a lack of time and the “mental burden” (participant 4) that this created for women. Participants often expressed that there were not enough hours in the day to do everything they felt was asked of them as caregivers, mothers, and clinician-scientists. Participants shared a common experience of finding themselves “stretched too thin” (participants 30 and 27) to simultaneously fulfill the high expectations of academia and gendered expectations of domestic responsibilities. The continual strain of constantly trying to balance between “work” and “life” and trying to calibrate their priorities created a substantial mental and emotional burden. Despite benefits like mandated paid leave for caregiving responsibilities, participants often felt that the culture of academic medicine was not accommodating. They reported feeling burdened with the need to justify taking leave both to colleagues and to themselves given the relentless pace and “work ethic” of the field. When recounting her experiences of child-rearing and academia, participant 14 recalled never feeling “unstressed, ever, for 1 minute….” For many women, particularly those with younger children, the COVID-19 pandemic exacerbated this already precarious balancing act. As participant 55 described when recounting her attempts to work and care for her children, “I worked way too much, and I fully burned myself out.”

Caregiving is typically thought of as an issue that primarily affects women in the early stages of their careers. However, most participants in our sample (17 individuals [54.8%]) reported having children who required adult supervision or care. Participants reported that mental and logistical demands of childrearing did not end once children gained more independence. Additionally, women perceived caregiving as coming with a “big hit to productivity” (participant 4) that could have long-lasting impacts on women’s career trajectories. Overall, caregiving was described as a responsibility that persists and continues to influence the availability of time and mental resources throughout women’s careers.

Participant 28 articulated that the emotional burden of time scarcity was heightened by the exhausting job of fighting bias and other systemic issues in academia. She described having “only…so much energy” to fight without making significant progress. Ultimately, this participant left academic medicine less than a year before this interview, citing a deep frustration and weariness with the field.

### Theme 2: Inequitable Treatment of Women in Bureaucratic Processes

Women also described the ways in which bureaucratic processes of academia disadvantaged them. They reported that these processes allowed many childrearing “hits to productivity” that women experienced during earlier phases of their career to persist through later career phases. They described challenges with getting back on track or staying on track in the first place due to inequities in the processes of resource allocation, grant application and award policies, and promotion and tenure.

Some women found themselves left out of important informational networks and excluded from policies that were “standard.” Participant 8 described an instance of not receiving funding that should have been standard, uncovering that other women were similarly excluded and then having to fight with her institution to get her and her colleagues what they were owed. She described how this situation “left a bad taste” in her mouth and harmed her trust in her institution. This situation also adversely impacted her productivity, she reported, given that she missed multiple years of administrative support that would have been available to her had she received funding on time.

Additionally, women described conflicts between rigid funding and promotion timelines and their caregiving responsibilities. Participant 17, whose child was facing serious health issues that demanded a leave of absence, experienced frustration when denied deferral of her K award. While she was given a no-cost extension as an alternative, she still functionally lost a year of work on her grant. She identified the lack of flexibility in grant timelines as her biggest career obstacle, a factor that disproportionately impacts women owing to their likelihood of encountering significant disruptions, such as childbearing and caregiving.

### Theme 3: Subtle and Less Subtle Professional Exclusion of Women

Women recounted instances of exclusion from professional and social circles within their institutions, which affected their access to power, mentorship, and professional advancement. For example, participant 28 was prevented from meeting with leadership without her mentor’s supervision; she was told, “that’s not the way we do it here.” She interpreted this as being taught that her role was to “sit down and shut up.” Conversely, men’s access to power was often protected. Participant 29 described an instance of a man who perpetrated sexual harassment and was allowed to remain in his position because he “got grants,” while women were told that their recourse was to not be in a room alone with him. A few women in our sample described these hostilities as coming from other women. For example, participant 57 reported that her mentor outwardly acted as though “she was all about women and promoting women” but took actions that created a toxic environment for other women. These instances of hostility served to create an environment where women felt unwelcome and to undermine their trust in their institutions.

Other women described ways in which they were “taught” that their voices were not welcomed at their institutions. Participant 1 described these subtle experiences as “some sort of ceiling above your head that you may not even realize is there…but is really shaping what you’re going to do with your career….” Participant 16 discussed how these invisible, gendered barriers can show up in mentoring relationships. She recalled a difference between the types of conversations men and women had with the same mentor, with men receiving guidance that was more informational and based on their scientific training, while women’s conversations were more centered on work and life balance. She perceived this as a way of communicating that childcare “should be a more important thing for you. You’re a woman.”

Women also described higher expectations to fulfill service-oriented roles (such as higher expectations to serve on committees, often without compensation or real authority) in their departments as a kind of professional exclusion. These roles simultaneously infringed on time that could be spent on research and were not recognized in promotion or tenure considerations. Participant 23 expressed frustration with being asked to, “do things simply because of who you are…not because of what you bring to the table.” Within these roles, women perceived that their presence was valued over their voice. This, coupled with the lack of recognition in promotion and tenure, further positioned women as “tokens” in the academic community instead of full participants. A disproportionate burden of representation in service-oriented roles fell most heavily on women in our sample who were members of a racial or ethnic minority group.

### Theme 4: Value of Communities Built on Shared Identities, Experiences, and Solidarity

When confronted with the various ways that academic medicine was biased against women, participants in our sample described sharing experiences with other women to be an important coping mechanism. Participant 28 shared how hearing similar experiences from other women helped her to feel less “isolated” and affirmed that she was not “crazy.” These communities were important in helping women realize that challenges they encountered were representative of system-level inequities rather than their own individual deficiencies. Other women also used their communities to advocate and identify ways to prevent future generations of women clinician-scientists from experiencing similar disadvantages.

These communities were often an important lifeline because they helped combat time scarcity. For example, participant 17 discussed the value of her group of female friends, emphasizing that they could confide in one another about work and life in their regular gatherings. Peer mentorship networks helped offer emotional and informational support related to childcare, while also providing “strategic advice” (participant 13) for navigating exclusions at work. Despite the benefits of these peer networks, some participants also noted the difficulty of finding and sustaining them, especially when institutional pilot programs ended.

## Discussion

This qualitative study found pervasive gendered burdens experienced by women clinician-scientists in academic medicine. These findings extend the current literature by describing gendered structures in academic medicine and how early-career experiences of gendered barriers persist into later career phases.

Participants in our study described how the dual pressure of childcare demands and rigorous demands of the ideal worker norm in academic medicine,^24,25^ an expectation of total dedication of time and prioritization of work, led to a feeling of “time scarcity.” This time scarcity led participants to experience high levels of stress and emotional distress when they felt that their identities as mothers and clinician-scientists were in conflict.

Our findings support prior research. Importantly, they highlight that challenges surrounding time scarcity identified in the early-career phase persist into the mid to senior phases^10^ given that most participants still had children who required supervision and that even adult children still required substantial parental investment. Caregiving-related demands, exacerbated by gendered unequal distribution of caregiving burden within heterosexual couples and norms of the ideal mother,^10,11^ can restrict women’s ability to engage fully in work activities or leverage opportunities for career advancement, particularly in fields with strong norms for ideal workers to focus on work to the exclusion of all else.^26,27^ To accommodate their dual responsibilities, women are more likely than men to work or consider working part time owing to the greater expectation of domestic labor at home,^28^ more likely to turn down opportunities for leadership and career advancement,^29^ and at greater risk of attrition.^30^ Women are also more likely to provide care for aging parents (their own and in-laws), such that they may have caregiving responsibilities in addition to their children and lingering responsibilities after children become more independent.^31^ For some participants, it was not until they approached retirement or left academic medicine that they were able to find respite from these pressures surrounding the prioritization of work over family. Many women in our sample were at a stage in their careers where they were not able to benefit from nascent family-friendly policies, and they described ways that early hurdles in productivity affected their trajectories.

Beyond time scarcity, women in our study also reported the mental burden of dealing with a “chilly climate” in academia.^19,32,33^ Our participants shared stories of systemic exclusion from professional and bureaucratic structures that underpin success in the field of academic medicine,^34^ including a lack of transparency in promotion and tenure processes,^9^ grant-funding mechanisms,^35^ and institutional resource allocation.^36^ Many participants indicated that caregiving and domestic responsibilities exacerbated these challenges by alienating the women from mentors or challenging their adherence to inflexible timelines. Several women described the additional time and mental burden they faced in experiencing and attempting to address systemic issues and the disproportionate burden, particularly for women in minority groups underrepresented in medicine, of service-oriented roles (roles requiring clinical, teaching, administrative, or other activities viewed as providing necessary service to others rather than independent scholarship or work primarily conducted to benefit the individual’s own career).^37^

Our study contributes to an increasing body of work on epistemic exclusion and professional marginalization faced in academia by women, particularly women in minority groups underrepresented in medicine.^34,37,38^ Given feelings of exclusion and a lack of accommodation, nearly all participants in our study expressed a deep frustration with a system that felt “broken.” While most participants described persisting in academic medicine despite these challenges, those who had left shared how this ultimately eroded their trust and enthusiasm for academic medicine. Participants described how their women mentors and peers validated their experiences, identified ways to navigate the biased system, and developed avenues for collective action, emphasizing that social support may be most powerful when anchored in shared experiences and positionalities.^39,40^ Initiatives that build such communities may help alleviate the mental burden that women in academia face and support their navigation of gendered institutional structures. It should be noted that while many women in our sample described mainly positive experiences with other woman peers and mentors, a few described instances of other women, mainly those in positions of authority, perpetuating a hostile climate rather than alleviating it. While bullying behavior in academic medicine is more likely to come from men,^41^ experiencing hostile behavior from other women can be particularly damaging. This suggests that interventions aimed at creating communities of support for women should also address ways that women can be strong allies for each other and change systems that perpetuate such behaviors.^42^

Given the challenges that mid- to senior-career women faculty face, there is a need for institutional efforts that address gender equity across the span of women’s professional trajectories. We caution readers against viewing these challenges as indicative of the ability or suitability of women or any caregivers to succeed in high-pressure environments. Our sample consists of women who all received competitive NIH awards and mostly persisted in academic medicine. Women in our study described systemic issues and established norms in the work environment that did not adequately account for or support workers with substantial personal commitments. These inequities must be addressed by redefining the outdated “ideal worker” model and implementing policies that center needs of caregivers regardless of gender.^7,43^

Our findings also suggest that the COVID-19 pandemic may have exacerbated already-existing challenges of caregiving burden, systemic exclusion, and difficulties identifying mentors and community. We echo concerns that the pandemic may have rolled back gains in women’s advancement.^44^ Additionally, while some institutions have taken steps to implement policies, processes, and programs to support early-career faculty with caregiving needs,^42^ institutions must continue to account for the differential impact that COVID-19 has had on women and other caregivers and the potential for long-lasting effects even after the acute impacts of the pandemic have lessened.

Some of this change is already underway. The NIH has implemented family-friendly policies that allow for inclusion of childcare costs in certain grant applications, reentry supplements for faculty who had to take time off for caregiving, and allowance to reduce effort to as low as three-quarter time.^45,46^ Other novel interventions aimed at reducing time burden, such as allowing faculty to earn credits for time spent in traditionally underrecognized service work, have shown positive results in increasing faculty grant attainment and relieving work-life stress. Still, more work is needed, especially to account for the ways in which early-career challenges may have lasting effects later in individuals’ careers and to develop enduring infrastructure to support women clinician-scientists beyond the early-career stage.

### Limitations

Our study findings should be considered within the context of their limitations. Notably, our sample of K awardees may not be representative of all women in academic medicine; those with particular interest in the topic may have been more likely to respond to our invitation. Still, we believe this cohort may offer especially pointed insights given study objectives. The timing of our interviews during the COVID-19 pandemic may have also impacted responses. Nevertheless, extant research has shown that the pandemic magnified previously existing concerns for women in academia. Our interviews mainly centered on career and workplace topics; although we did not analyze or probe partner status, it is likely that the experience of the caregiving time burden is affected by relationship status. Additionally, that our respondents described experiencing academic medicine as “centered around male[s]” should not be interpreted to mean that academic medicine is hospitable for all men. Many challenges described by women in this study also apply to men who deviate from traditional gendered roles and ideal worker norms, speaking to broader needs to accommodate family caregiving.

## Conclusions

In this qualitative study, mid- to senior-career women clinician-scientists in academic medicine perceived being disadvantaged by their gender over the course of their careers. They reported that this occurred through the confluence of domestic obligations and institutional environments that excluded them or made it difficult to get back on track if they deviated from established timelines or norms of career progression and productivity. Despite high rates of attrition of women in academic medicine early in their careers, even those who persevered in the face of these obstacles reported continuing to be impacted by these same burdens. Acknowledging these challenges that midcareer and senior women face, our findings suggest that institutions must consider initiatives that address structural elements in policies and processes of academia that exclude and disadvantage women and others with family responsibilities.

## Notes

Supplement.
Data Sharing Statement
