# Supplementary material for: Professional Experiences and Career Trajectories of Mid- to Senior-Career Women Clinician-Scientists: A Qualitative Study
Source: JAMA Netw Open. 2024 Apr 11;7(4):e246040. doi: 10.1001/jamanetworkopen.2024.6040 (PMC11253288; doi:10.1001/jamanetworkopen.2024.6040)
Supplement: Supplement. — Data Sharing Statement [file jamanetwopen-e246040-s001.pdf]

Data Sharing Statement

Szczygiel. Professional Experiences and Career Trajectories of Mid to Senior Career Women Clinician-Scientists: A Qualitative Study. *JAMA Netw Open*. Published online Apirl 11, 2024. doi:10.1001/jamanetworkopen.2024.6040

Data
Data available: No

Additional Information
Explanation for why data not available: Data in this study will not be shared to protect participant confidentiality.
